# Effects of cilostazol on renal function, renal blood flow, and long-term prognosis in patients with chronic kidney disease

**DOI:** 10.1007/s10157-026-02897-8

**Published:** 2026-06-08

**Authors:** Chie Saito, Kaori Mase, Shun Ishibashi, Takuya Harada, Ryoya Tsunoda, Toshiaki Usui, Tatsuya Simizu, Kei Nagai, Reiko Ohkubo, Naoki Morito, Masahiro Hagiwara, Hirayasu Kai, Joichi Usui, Kunihiro Yamagata

**Affiliations:** Department of Nephrology, Institute of Medicine, University of Tsukuba, Tsukuba, Japan

**Keywords:** Cilostazol, Glomerular filtration rate, Estimated GFR, Long-term effect, Renal replacement therapy initiation

## Abstract

**Background:**

Cilostazol has antiplatelet and vasodilatory properties and may provide renoprotective and cardiovascular benefits; however, its effects in patients with advanced chronic kidney disease (CKD) remain unclear.

**Methods:**

This prospective pilot study enrolled 12 patients with advanced CKD due to nephrosclerosis (7 men, 5 women; mean age 65.3 ± 11.0 years). Patients were randomly assigned to receive cilostazol 200 mg/day plus aspirin 100 mg/day (Group C, n = 6) or aspirin 100 mg/day alone (Group A, n = 6). Glomerular filtration rate (GFR) and renal plasma flow (RPF) were measured using inulin and para-aminohippuric acid clearance at baseline and after 1 year. Renal outcomes and complications were evaluated, followed by long-term observation until initiation of renal replacement therapy (RRT).

**Results:**

Baseline GFR and RPF were comparable between groups. After 1 year, GFR increased in 4 of 6 patients in Group C but declined in all patients in Group A. Mean GFR decreased significantly in Group A but not in Group C. Although estimated GFR declined in both groups, the annual rate of decline was significantly slower in Group C than in Group A (− 2.72 vs. − 3.84 mL/min/1.73 m^2^/year). RPF and urinary protein levels showed no significant changes in either group. Over a mean follow-up of 6.7 years, RRT was initiated in 2 patients in Group C and 4 in Group A. Two cerebral infarctions occurred in Group A, whereas no cardiovascular events occurred in Group C.

**Conclusion:**

Cilostazol may slow short-term renal function decline and offer long-term cardiovascular protection in patients with advanced CKD. Further studies are needed to determine its role in preventing long-term renal deterioration.

## Introduction

Antiplatelet agents, due to vasodilatory and antithrombotic effects, are effective in preventing the onset and recurrence of atherosclerotic occlusive disease, ischemic heart disease, and cerebral infarction, with a demonstrated efficacy in large, prospective studies. Their therapeutic utility is particularly well-established for cerebral infarction and atherosclerotic occlusive disease [1]. Among these agents, cilostazol possesses the strongest evidence base for preventing cardiovascular disease (CVD) events [2].

Meanwhile, chronic kidney disease (CKD) has recently gained significant recognition, driven by global aging trends, an increasing number of end-stage renal failure patients requiring renal replacement therapy, and its recognition as a major risk factor for CVD mortality [3]. Antiplatelet agents are traditionally prescribed for CKD to reduce proteinuria and ablate functional decline, with phosphodiesterase (PDE) inhibitors reported to have specific renoprotective effects, such as suppressing albuminuria (in diabetic nephropathy) [4], proteinuria (in glomerulonephritis) [5], and deterioration in renal function [6]. However, large-scale, randomized, controlled trials verifying their efficacy remain unreported.

This study randomly assigned Stage G3 and G4 CKD patients to receive either cilostazol plus aspirin or aspirin only. It evaluated detailed changes in renal function around the time of administration, including glomerular filtration rate (GFR) (inulin clearance) and renal plasma flow (RPF) (para-aminohippuric acid clearance). Long-term follow-up was then conducted to examine the long-term effects of the antiplatelet agent on renal function impairment and the incidence of CVD.

## Methods

This study was a prospective randomized controlled pilot study conducted at the University of Tsukuba, Japan, from 2007 to 2009. The participants were 12 patients (7 males, 5 females, mean age 65.3 ± 11.0 years) with reduced renal function corresponding to CKD stages G3 and G4, i.e., estimated glomerular filtration rate (eGFR) ≥ 15 mL/min/1.73 m^2^ and < 60 mL/min/1.73 m^2^, and negative to < 1 g/day proteinuria (Table 1). Patients were assigned alternately to Group C or Group A in order of enrollment (open-label design; UMIN000000527). Primary outcomes were changes in GFR and RPF at 1 year. Secondary/safety outcomes, including eGFR slope, time to RRT initiation, and cardiovascular events (including cerebrovascular disease), were prespecified at study initiation. GFR, RPF, and eGFR were measured before treatment and after 1 year of treatment.

**Table 1:** Baseline characteristics of study participants

Variable	Total (N = 12)	Group C (N = 6)	Group A (N = 6)	p value
*Demographics*				
Age (yr)	66.5 (58.2–73.2)	64.5 (55.2–73.8)	66.5 (65.0–71.8)	0.63
Male / Female	7 / 5	4 / 2	3 / 3	–
Body weight (kg)	60.0 (51.0–64.1)	62.3 (50.2–65.4)	58.3 (52.7–61.4)	0.82
BMI (kg/m^2^)	24.5 (22.4–27.6)	24.2 (19.8–28.2)	24.8 (22.8–26.7)	0.94
*Comorbidities and risk factors*				
Hypertension	10/12	4/6	6/6	–
Diabetes mellitus (type 2)	2/12	1/6	1/6	–
Dyslipidemia	7/12	4/6	3/6	–
Smoking (current or former)	2/12	1/6	2/6	–
*Hemodynamics*				
Systolic BP (mmHg)	135.0 (127.5–140.0)	135.0 (128.5–140.0)	135.0 (124.0–145.2)	0.87
Diastolic BP (mmHg)	71.0 (65.5–75.5)	74.0 (74.0–78.5)	67.5 (61.5–71.2)	0.06
Heart rate (bpm)	72.5 (66.0–80.0)	72.0 (67.5–78.0)	74.0 (65.8–88.2)	0.69
*Renal function at baseline*				
s-Creatinine (mg/dL)	1.6 (1.3–2.4)	1.3 (1.3–2.0)	2.1 (1.4–2.5)	0.82
eGFR (mL/min/1.73 m^2^)	28.6 (22.6–36.2)	34.4 (26.0–38.7)	23.0 (22.1–32.0)	0.42
GFR-inulin (mL/min/1.73 m^2^)	27.1 (21.4–35.0)	27.1 (24.7–31.3)	24.6 (20.9–38.4)	0.94
RPF-PAH (mL/min/1.73 m^2^)	172.8 (118.2–208.7)	194.7 (160.0–223.9)	142.8 (114.8–176.4)	0.31
Filtration fraction	0.16 (0.14–0.19)	0.14 (0.13–0.17)	0.18 (0.17–0.20)	0.14
Proteinuria (g/day)	0.40 (0.11–0.78)	0.21 (0.09–0.41)	0.57 (0.22–1.10)	0.48
Serum albumin (g/dL)	4.0 (3.9–4.2)	3.9 (3.9–4.0)	4.2 (4.0–4.3)	0.22
*Medications*				
Antihypertensives	10/12	4/6	6/6	–
Statins	3/12	2/6	1/6	–
SGLT2 inhibitors	0/12	0/6	0/6	–

Values are expressed as median (interquartile range) for continuous variables, or n/N for categorical variables

Between-group comparisons by Mann–Whitney U test

*BMI* body mass index, *BP* blood pressure, *bpm* beats per minute, *eGFR* estimated glomerular filtration rate, *FF* filtration fraction, *GFR* glomerular filtration rate, *RPF* renal plasma flow, *SGLT2* sodium-glucose cotransporter-2

No patients received SGLT2 inhibitors (study conducted 2007–2009)

Dyslipidemia: based on comorbidity data. Smoking: current or former smoker

GFR was measured using inulin clearance, and RPF was measured using para-aminohippuric acid clearance. Details of the measurement methods were previously published [7]. Nutritional guidance and lifestyle guidance were provided to all patients immediately before starting treatment.

Follow-up was conducted with an endpoint of end-stage chronic renal failure or death, targeting approximately 10 years after the study initiation, to prospectively observe renal function and the occurrence of complications such as CVD. Renal function after the first year was assessed by calculating eGFR based on serum creatinine and evaluating its change over time.

### Statistical analysis

Normality of continuous variables was assessed using the Shapiro–Wilk test. Continuous variables are presented as median (interquartile range [IQR]). For comparisons between two groups, the Mann–Whitney U test was used. For changes in GFR, RPF, and eGFR over time, a two-way repeated-measures analysis of variance (ANOVA) was used to assess the interaction between time (baseline vs. 1 year) and treatment group. Survival analysis was performed using the Kaplan–Meier method, and comparisons between two groups used the log-rank test. The significance level was set at p < 0.05. Statistical analysis was performed using the SPSS software program version 27 (Armonk, NY: IBM Corporation).

### Ethical considerations

This study was initiated after approval by the Ethics Committee of the University of Tsukuba Hospital (Approval No. H18-68). Written, informed consent was obtained from all participants after explanation of the study details. This study was registered in the University hospital Medical Information Network (UMIN) Center database (https://www.umin.ac.jp) as UMIN000000527.

## Results

### Changes in GFR, RPF, and eGFR before and after cilostazol administration

**Fig. 1:**
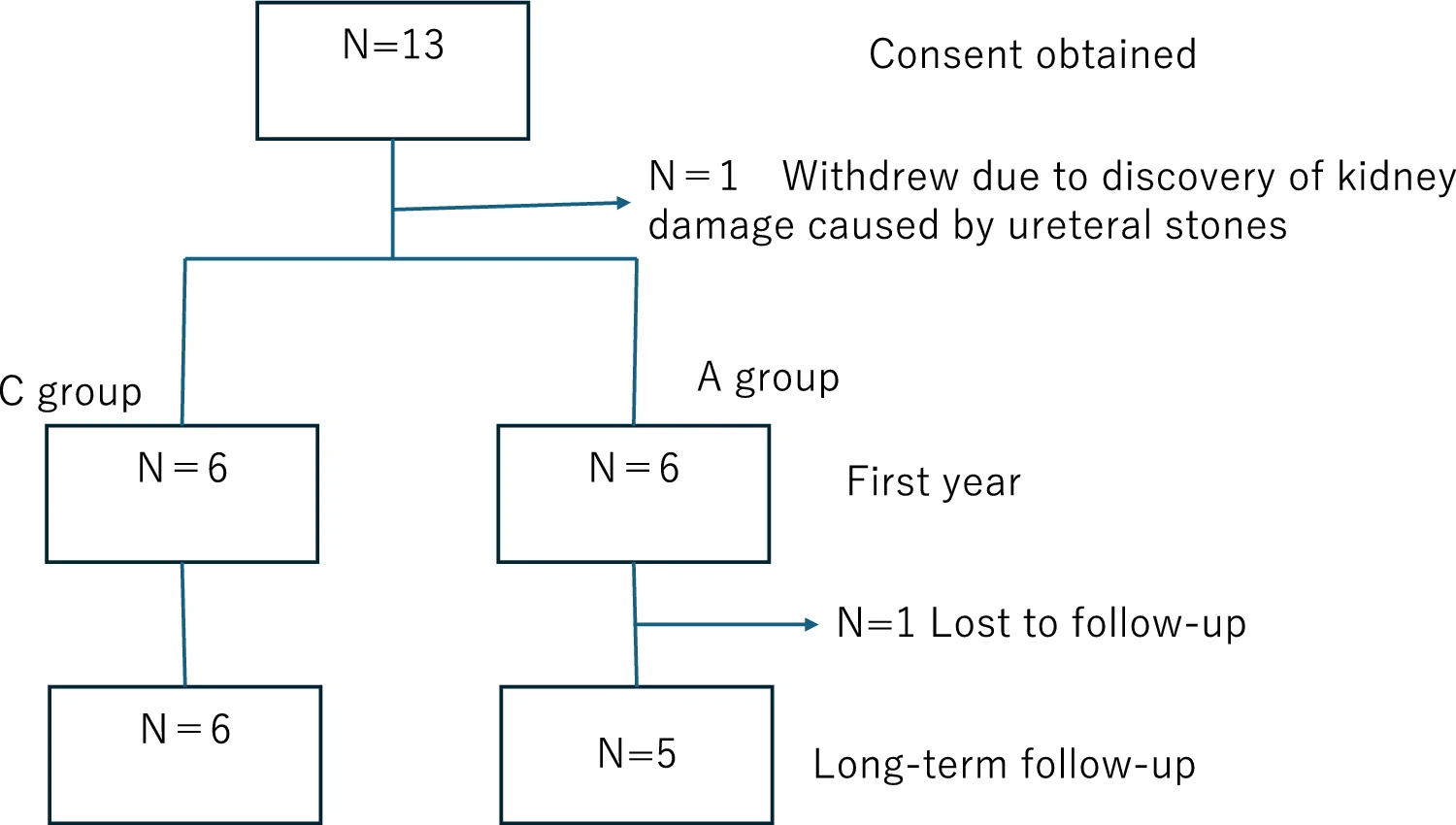
Subjects. Thirteen patients were recruited, but one was excluded as their renal impairment was diagnosed as being caused by urinary stones prior to the start of this trial. The 12 remaining patients were randomly divided into two groups. After confirming detailed changes in renal function over approximately one year, one patient in Group A was lost to follow-up due to transfer to another hospital; consequently, the full course of the study could be followed for 11 patients

A total of 13 patients were recruited but one was excluded after consent acquisition due to renal function decline caused by hydronephrosis from urinary calculi. Thus, a total of 12 patients (7 males, 5 females, mean age 65.3 ± 11.0 years) with reduced renal function corresponding to CKD stages G3 and G4, i.e., estimated glomerular filtration rate (eGFR) ≥ 15 mL/min/1.73 m^2^ and < 60 mL/min/1.73 m^2^, and negative to < 1 g/day proteinuria were enrolled (Fig. 1). Patient characteristics and baseline data are summarized in Table 1. Hypertension was present in 80% of Group C and 100% of Group A. Diabetes (Type 2) was present in 16.7% of both groups. One participant assigned to Group C was excluded from long-term follow-up after 1-year renal function testing due to transfer to another hospital (Fig. 1).

**Fig. 2:**
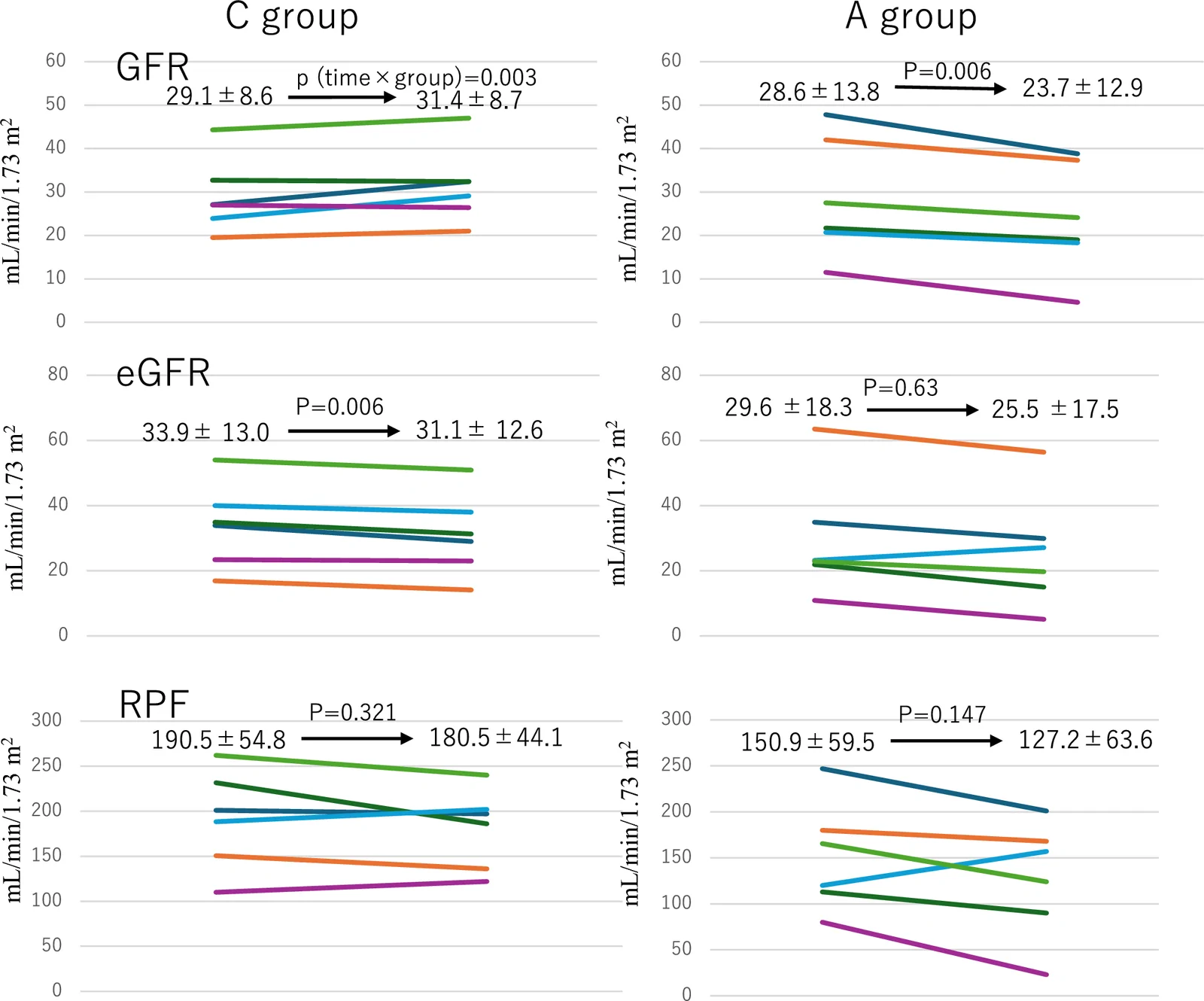
One-year changes in renal function of the subjects. This figure shows a comparison of GFR, eGFR and RP before intervention and 1 year later. In Group C, GFR increased in four cases and showed a mild decrease in two cases; however, in Group A, GFR decreased in all cases, showing a significant decrease between the pre- and post-intervention periods. eGFR showed a significant decrease in Group B. There were no changes in RPF in either group between the pre- and post-intervention periods

There were no significant differences between groups in baseline GFR, RPF, or degree of proteinuria. After 1 year of intervention, GFR increased in 4 of 6 participants in Group C, while it decreased in all participants in Group A. The mean GFR increased in Group C from 29.1 ± 8.6  to 31.4 ± 8.7 ml/min/1.73 m^2^, while it decreased significantly in Group A from 28.6 ± 13.8  to 23.7 ± 12.9 ml/min/1.73 m^2^. In contrast, while eGFR decreased in all cases in Group C, it increased in one case and decreased in the other five cases in Group A, with a larger average value decrease in Group A (Fig. 2). Comparing the annual speed of renal function change over 1 year, GFR improved by 2.25 ml/min/1.73 m^2^/year in Group C, while it worsened by – 4.7 ml/min/1.73 m^2^ in Group A, showing a significant difference between the two groups. The annual average deterioration speed of eGFR was – 2.72 ml/min/1.73 m^2^/year in Group C and – 3.84 ml/min/1.73 m^2^/year in Group A, showing a significantly slower deterioration speed in Group C. There were no significant changes in RPF or urinary protein in either group (Table 2) and no CVD events in either group was observed during the first year.

**Table 2:** Renal function slope and proteinuria changes

Variable (mL/min/1.73 m^2^/year unless noted)	Group C (N = 6)	Group A (N = 6)	p value
*Year 1 changes*			
GFR (inulin) slope	+ 2.03 (0.13–4.48)	− 3.86 (− 6.30 to − 2.55)	0.002
eGFR slope	− 2.84 (− 3.43 to − 2.17)	− 5.37 (− 6.01 to − 3.39)	0.18
RPF slope	− 8.85 (− 19.53–7.69)	− 28.70 (− 43.69 to − 14.07)	0.39
Proteinuria change (Δg/day)	+ 0.02 (− 0.09 to 0.10)	+ 0.02 (− 0.05–0.13)	0.70
*Total follow-up changes*			
eGFR slope (entire follow-up)	− 2.61 (− 2.84 to −2.30)	− 3.07 (− 3.69– to − 1.64)	1.00
Proteinuria change total (Δg/day)	+ 1.09 (0.18–1.38)	+ 0.32 (− 0.01 to 0.98)	0.79

Values are expressed as median (interquartile range). Between-group comparisons by Mann–Whitney U test

*eGFR* estimated glomerular filtration rate, *GFR* glomerular filtration rate, *RPF* renal plasma flow

GFR slope calculated from inulin clearance (baseline vs. 1 year). Total follow-up eGFR slope calculated over entire observation period

Time × group interaction for GFR by two-way repeated-measures ANOVA: *p* = 0.003

### Long-term follow-up results

**Fig. 3:**
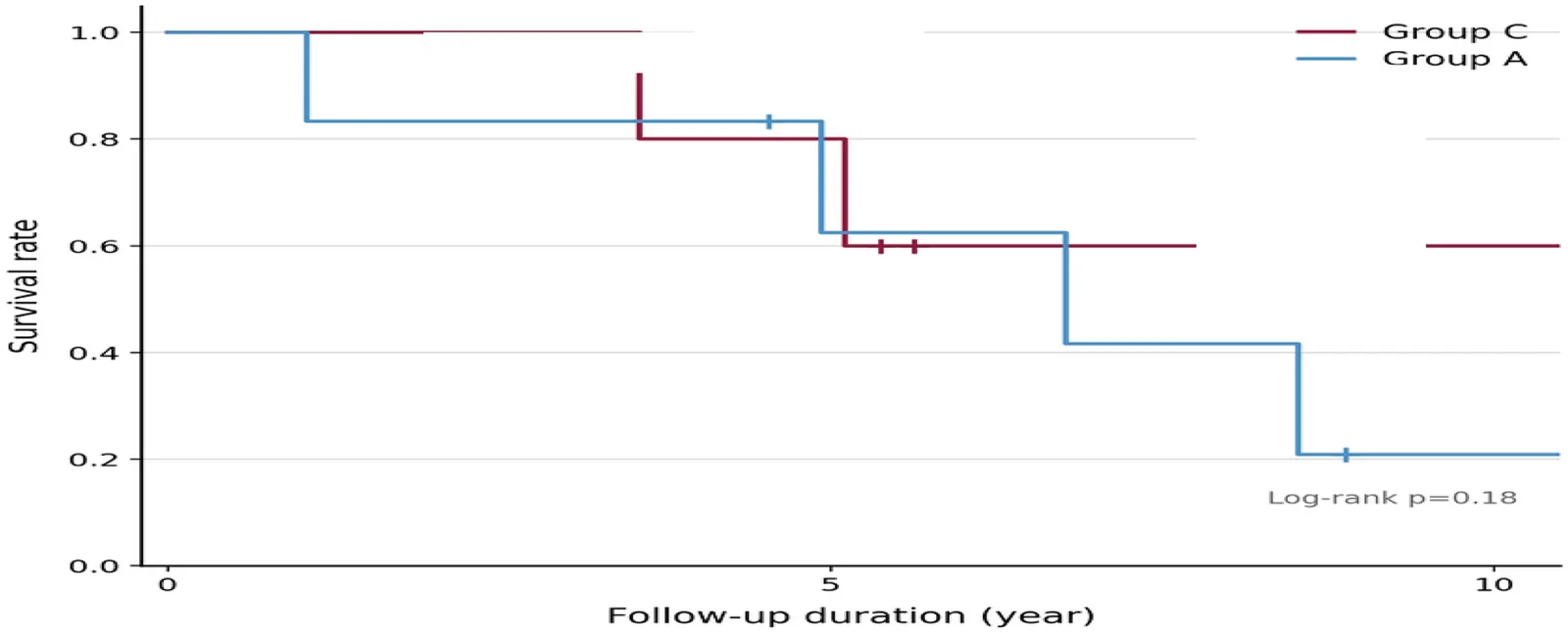
Kaplan–Meier survival curve of the subjects. In the results of long-term follow-up, with the initiation of renal replacement therapy as the endpoint, there were fewer cases reaching this endpoint in Group C, but there was no significant difference between the two groups

Intention-to-treat follow-up analysis was performed up to 10 years for each enrolled patient. The median follow-up duration was 6.8 years (IQR: 4.9–8.5 years). During this period, renal replacement therapy was initiated in 2 patients in Group C and 4 patients in Group A but Kaplan–Meier analysis of renal prognoses showed no significant differences between groups (Fig. 3). The mean annual rate of eGFR decline was also not significantly different between groups; however, over the 10-year follow-up, Group C tended to reach end-stage CKD later. Both groups showed an increase in proteinuria during the long-term observation period.

### Adverse events and safety profile of cilostazol

During the initial one-year period of this study, no new cases of CVD occurred in either group, nor were any bleeding events or other adverse effects observed. During the long-term follow-up period, one patient in Group C developed a hemorrhagic gastric ulcer confirmed by endoscopy (October 2009), which may have been related to antiplatelet therapy. Additionally, during the long-term follow-up period, three new cerebral infarctions occurred in Group A while one new cerebral infarction was observed in Group C but these were not linked to cilostazol-mediated adverse events.

## Discussion

CKD is rapidly increasing due to population aging, a rising prevalence of lifestyle-related diseases, and environmental changes. Consequently, the global increase in patients requiring renal replacement therapy due to end-stage kidney disease and the associated rise in healthcare costs have become significant public health issues [8]. Furthermore, as CKD is a major risk factor for CVD onset, CKD treatment has gained attention for prevention/progression of CVD, which remains a leading cause of global mortality [9]. Guidelines to suppress the progression of CKD and preserve renal function have resulted in increasing use of RAS inhibitors [10], SGLT2 inhibitors [11], aldosterone antagonists [12, 13], and GLP-1 agonists [14] to suppress both proteinuria and renal function deterioration. Meanwhile, antiplatelet agents with phosphodiesterase (PDE) inhibitory effects may support kidney hemodynamics by preserving the phosphodiester bond in the CREB/cAMP/cGMP second messenger system, protecting glomerular integrity, increasing angiogenesis, boosting antioxidant/anti-apoptosis function, and preventing fibrosis in addition to reducing chronic inflammation[15]. In light of this strong supporting effect, recent momentum exists for re-evaluating their efficacy in CKD [4, 6].

We thus prospectively investigated the detailed effects of cilostazol, an antiplatelet agent with PDE inhibitory activity and recognized evidence for preventing CVDs, particularly cerebral infarction, on renal function, renal blood flow, and renal prognosis over a 10-year follow-up period after initiation. Our results showed that cilostazol administration could maintain or improve GFR for at least one year. We also observed increased renal survival over the long-term follow-up period. Beneficial results were also noted by other cilostazol studies in hemodialysis patients (improved survival up to ~ 6 years later) [16] and patients at risk of recurrent ischemic stroke [17] but a key point to note is the divergence we observed in GFR between GFR fluctuations and eGFR (cre) fluctuations. GFR (i.e., inulin clearance) measures true GFR, whereas eGFR is an estimated value based on serum creatinine levels and age, assuming a decline in renal function due to aging during follow-up. Therefore, even if there are no changes in body composition, true GFR is maintained, and serum creatinine levels remain unchanged, the eGFR value will inevitably decrease after 1 year due to these assumptions in the calculation. This is considered responsible for the temporal changes of GFR and eGFR in the cilostazol group since, in the temporal analysis comparing the cilostazol-treated group and the non-cilostazol-treated group, the cilostazol-treated group showed a clear suppression of decline in both GFR and eGFR. This suppression of renal function decline was evident, not merely maintaining measured GFR but actually improving it. Furthermore, since there was no difference in RPF changes between the two groups, the immediate post-treatment improvement in GFR is thought to result from cilostazol-induced reduction in glomerular capillary pressure stemming from a decrease in glomerular filtration pressure. Thus, we hypothesize that cilostazol has a primary effect due to prevention of glomerular injury due to preservation of pressure homeostasis.

Although useful for global downregulation of prostaglandin-induced inflammation, reports of the effects of aspirin on renal function have ambiguous results. While some studies, including a study in 11,846 patients from the Therapeutic Interventions in Chronic Kidney Disease (TRI-CKD) cohort, reported low-dose aspirin use as significantly associated with long-term decreases in renal function, other, large analyses report no effect [18, 19]. It has been suggested that the inhibition of prostaglandins PGE₂ and PGI₂ leads to reduced cortical blood flow, decreased glomerular filtration pressure, and, consequently, reduced GFR [20]. Although the effects of antiplatelet agents on GFR in CKD patients have not been fully investigated, epidemiological studies report that administering high-dose antiplatelet agents, as used in CKD, may have a long-term protective effect against worsening renal function [20]. Among these, cilostazol is known to reduce glomerular pressure by alleviating oxidative stress and modulating TGF-β and NF-κB expression [21] while exerting vasodilatory and antiproliferative effects via cAMP increase mediated by PDE3 inhibition [22]. These mechanisms are thought to contribute to the maintenance of renal function under cilostazol administration and may be a key point of beneficial effects seen in studies of kidney health in CVDs [23].

Furthermore, among the four drug classes recently expected to improve CKD prognosis—RAS inhibitors, SGLT2 inhibitors, aldosterone antagonists, and GLP-1 agonists—an initial dip in GFR has been reported with all of these agents, including GLP-1 receptor agonists as demonstrated in the SELECT trial [24]. Regarding this initial dip in GFR, many reports suggest that, as the subsequent rate of renal decline improves, long-term renal prognosis may be expected to improve [25, 26]. However, previous studies have not included sufficient long-term follow-up and this initial dip is notably not observed in all cases; reports also indicate that patients exhibiting an initial dip may have poorer renal outcomes and could be an as-yet unelucidated subgroup with a specific phenotype that renders them GFR-sensitive [27, 28].

In this context, the addition of cilostazol may suppress early renal decline, potentially offering long-term renal protection alongside CVD prevention, including stroke. In this study, 4 of the 6 patients who started cilostazol were already on RAS inhibitors, but none were newly prescribed these agents. Future research should address whether concomitant use of antiplatelet agents with RAS inhibitors or SGLT2 inhibitors suppresses the initial dip, as well as the progression of renal function deterioration and the occurrence of CKD-related complications in such patients. In recent years, the increasing elderly population in developed nations has made the rising burden of public health costs a critical issue. Furthermore, there has been an increasing trend where lower-cost existing drugs become unprofitable for pharmaceutical companies, leading to discontinuation and antiplatelet agents fall into this category. However, they show no inferiority to existing drugs in improving the long-term prognosis of CKD and may additionally contribute to improving the prognosis of CKD patients, suggesting the need for re-evaluation.

This study has limitations, including a small number of participants, the inability to evaluate the effect of cilostazol on the initial dip due to not initiating it concurrently with RAS inhibitors, the lack of histopathological diagnosis of renal impairment directly related to CKD prognosis, and the absence of natriuretic peptide levels and echocardiographic data, limiting formal assessment of cilostazol-related cardiac safety. Formal covariate adjustment for baseline differences between groups was not statistically feasible given the very small sample size. Nevertheless, this study suggests that in patients with age-related renal impairment, cilostazol administration may offer safe, short-term improvement in glomerular filtration rate and, long-term, potentially delay progression to ESKD and suppress the onset of CVD.

## Data Availability

Data sharing statement The datasets used in the current analysis are available from the corresponding author upon reasonable request.

## References

[1] Leonardi-Bee J, et al. Dipyridamole for preventing recurrent ischemic stroke and other vascular events: a meta-analysis of individual patient data from randomized controlled trials. Stroke. 2005;36(1):162–8. 15569877 10.1161/01.STR.0000149621.95215.ea

[2] Manolis AA, et al. Update on cilostazol: a critical review of its antithrombotic and cardiovascular actions and its clinical applications. J Clin Pharmacol. 2022;62(3):320–58. 34671983 10.1002/jcph.1988

[3] Levey AS, et al. National Kidney Foundation practice guidelines for chronic kidney disease: evaluation, classification, and stratification. Ann Intern Med. 2003;139(2):137–47. 12859163 10.7326/0003-4819-139-2-200307150-00013

[4] Tang WH, et al. Cilostazol effectively attenuates deterioration of albuminuria in patients with type 2 diabetes: a randomized, placebo-controlled trial. Endocrine. 2014;45(2):293–301. 23775007 10.1007/s12020-013-0002-3

[5] Tojo S, et al. Dipyridamole therapy in the nephrotic syndrome. Contrib Nephrol. 1978;9:111–27. 352614 10.1159/000401438

[6] Kuo KL, et al. Dipyridamole decreases dialysis risk and improves survival in patients with pre-dialysis advanced chronic kidney disease. Oncotarget. 2018;9(4):5368–77. 29435184 10.18632/oncotarget.19850 PMC5797055

[7] Matsuo S, et al. Revised equations for estimated GFR from serum creatinine in Japan. Am J Kidney Dis. 2009;53(6):982–92. 19339088 10.1053/j.ajkd.2008.12.034

[8] G.B.D.C.K.D. Collaboration. Global, regional, and national burden of chronic kidney disease, 1990-2017: A systematic analysis for the Global Burden of Disease Study 2017. Lancet. 2020;395(10225):709–33. 32061315 10.1016/S0140-6736(20)30045-3 PMC7049905

[9] Hannan M, et al. Risk factors for CKD progression: overview of findings from the CRIC study. Clin J Am Soc Nephrol. 2021;16(4):648–59. 33177074 10.2215/CJN.07830520 PMC8092061

[10] Brenner BM, et al. Effects of losartan on renal and cardiovascular outcomes in patients with type 2 diabetes and nephropathy. N Engl J Med. 2001;345(12):861–9. 11565518 10.1056/NEJMoa011161

[11] Heerspink HJL, et al. Dapagliflozin in patients with chronic kidney disease. N Engl J Med. 2020;383(15):1436–46. 32970396 10.1056/NEJMoa2024816

[12] Agarwal R, et al. Finerenone with Empagliflozin in Chronic Kidney Disease and Type 2 Diabetes. N Engl J Med. 2025;393(6):533–43. 40470996 10.1056/NEJMoa2410659

[13] Bakris GL, et al. Effect of Finerenone on chronic kidney disease outcomes in type 2 diabetes. N Engl J Med. 2020;383(23):2219–29. 33264825 10.1056/NEJMoa2025845

[14] Badve SV, et al. Effects of GLP-1 receptor agonists on kidney and cardiovascular disease outcomes: a meta-analysis of randomised controlled trials. Lancet Diabetes Endocrinol. 2025;13(1):15–28. 39608381 10.1016/S2213-8587(24)00271-7

[15] Thapa K, Singh TG, Kaur A. Cyclic nucleotide phosphodiesterase inhibition as a potential therapeutic target in renal ischemia reperfusion injury. Life Sci. 2021;282:119843. 34298037 10.1016/j.lfs.2021.119843

[16] Lim PS, et al. Role of cilostazol therapy in hemodialysis patients with asymptomatic peripheral arterial disease: a retrospective cohort study. Biomed Res Int. 2016;2016:8236903. 27747241 10.1155/2016/8236903 PMC5055930

[17] Koge J, et al. Effect of renal function on dual antiplatelet therapy using cilostazol for stroke prevention: A CSPS.com trial post hoc analysis. J Neurol Sci. 2025;477:123661. 40845415 10.1016/j.jns.2025.123661

[18] Lu JL, et al. Association of long-term aspirin use with kidney disease progression. Front Med Lausanne. 2023;10:1283385. 38111701 10.3389/fmed.2023.1283385 PMC10726126

[19] Eskandarian R, Pari SM, Memarian M. Examining the impact of aspirin on patients with chronic kidney disease; a systematic review and meta-analysis. J Ren Inj Prev. 2024. 10.34172/jrip.2024.37317.

[20] Bonet-Monne S, et al. NSAIDs, analgesics, antiplatelet drugs, and decline in renal function: a retrospective case-control study with SIDIAP database. BMC Pharmacol Toxicol. 2024;25(1):58. 39198874 10.1186/s40360-024-00771-5 PMC11351315

[21] Lee WC, et al. Cilostazol ameliorates nephropathy in type 1 diabetic rats involving improvement in oxidative stress and regulation of TGF-Beta and NF-kappaB. Biosci Biotechnol Biochem. 2010;74(7):1355–61. 20622454 10.1271/bbb.90938

[22] Kherallah RY, et al. Cilostazol: a review of basic mechanisms and clinical uses. Cardiovasc Drugs Ther. 2022;36(4):777–92. 33860901 10.1007/s10557-021-07187-x

[23] Wu CK, et al. Long-term effectiveness of Cilostazol in patients with hemodialysis with peripheral artery disease. J Atheroscler Thromb. 2023;30(8):943–55. 36216573 10.5551/jat.63404 PMC10406651

[24] Colhoun HM, et al. Long-term kidney outcomes of semaglutide in obesity and cardiovascular disease in the SELECT trial. Nat Med. 2024;30(7):2058–66. 38796653 10.1038/s41591-024-03015-5 PMC11271413

[25] Bakris GL, Weir MR. Initial drops in glomerular filtration rate with certain drug classes retard kidney disease progression. Am J Nephrol. 2022;53(7):513–5. 35691290 10.1159/000524890

[26] Kawano R, et al. Favorable changes in the eGFR slope after dapagliflozin treatment and its association with the initial dip. Clin Exp Nephrol. 2024;28(12):1282–9. 38970649 10.1007/s10157-024-02532-4

[27] Schmidt M, et al. Serum creatinine elevation after renin-angiotensin system blockade and long term cardiorenal risks: cohort study. BMJ. 2017;356:j791. 28279964 10.1136/bmj.j791 PMC5421447

[28] Kanaoka T, et al. Factors affecting the sodium-glucose cotransporter 2 inhibitors-related initial decline in glomerular filtration rate and its possible effect on kidney outcome in chronic kidney disease with type 2 diabetes: The Japan Chronic Kidney Disease Database. Diabetes Obes Metab. 2024;26(7):2905–14. 38719436 10.1111/dom.15611

